# Masturbation parameters related to orgasm satisfaction in sexual relationships: Differences between men and women

**DOI:** 10.3389/fpsyt.2022.903361

**Published:** 2022-07-22

**Authors:** Oscar Cervilla, Juan Carlos Sierra

**Affiliations:** Mind, Brain, and Behavior Research Center (CIMCYC), University of Granada, Granada, Spain

**Keywords:** orgasm satisfaction, partnered sex, masturbation, subjective orgasm experience, attitude toward masturbation, sex differences

## Abstract

**Objective:**

Masturbation is a behavior that can enhance sexual functioning. This study aims to analyze differences between men and women in different masturbation parameters, and to examine their relation with orgasm satisfaction in sexual relationships.

**Method:**

One thousand three hundred and thirty-fifth men and women from the Spanish population aged 18–83 years (*M* = 36.91; *SD* = 11.86) participated in an online survey. A questionnaire was used to collect socio-demographic. Sexual history data, negative attitude toward masturbation, solitary sexual desire and orgasm subjective experience upon masturbation were assessed. Given the differences between men and women, independent regression models are proposed to explain orgasm satisfaction in the sexual relationships context.

**Findings:**

Men, compared to women, masturbated at a younger age (*p* < 0.001), and reported higher current masturbation frequency (*p* < 0.001) and more solitary sexual desire (*p* < 0.001). Women reported greater intensity in the subjective orgasm experience on its Affective (*p* < 0.001), Sensory (*p* < 0.001) and Intimacy (*p* < 0.001) dimensions. Regarding regression models, the Affective dimension of orgasm was a common parameter in men (β = 0.36; *p* < 0.001) and women (β = 0.24) to explain orgasm satisfaction during sexual relationships. In men, solitary masturbation frequency (β = −0.10; *p* = 0.027) acquired a significant role. In women, the model also included age (β = 0.09; *p* = 0.038), negative attitude toward masturbation (β = −0.12; *p* = 0.005) and solitary sexual desire (β = −0.19; *p* = 0.001).

**Conclusion:**

When dealing with men and women's orgasm difficulties in the sexual relationships context, it is important to consider the role of masturbation. In men and women, the Affective dimension of the orgasm experience explain the orgasm satisfaction in sexual relationship. Also, in men, the solitary masturbation frequency is negatively related with orgasm satisfaction in sexual relationship, supporting the compensatory hypothesis of masturbation. In women, in addition to the Affective dimension, the orgasm satisfaction in sexual relationship is explained, negatively, by the negative attitude toward masturbation, and positively, by the solitary sexual desire, which could be associated with more sexual self-knowledge. The relevance of masturbation in understanding sexual functioning is highlighted.

## Introduction

Masturbation is a relatively frequent behavior that is positively associated with sexual health (1–5). More importance has been attached to study it in recent decades, and its capacity to promote sexual self-knowledge and to elicit positive sexual responses has been underlined (6, 7). Among these good points, its usefulness in sexual therapy to improve sexual functioning has been stressed (8).

Very little evidence exists for the relation between masturbation and sexual relationships (9). The association between both sexual behaviors has been described by two models: compensatory vs. complementary. The former proposes practicing masturbation to replace desired sexual relationships that do not take place (10, 11). The fact that higher masturbation frequency is related to lower sexual satisfaction, and has been found for women, favors this model (12), while higher masturbation frequency for those who less enjoy their sexual relationships has been described for men (13). The complementary model hypothesizes that a direct positive relation exists between both these sexual activities, and increasing the practice of one would be associated with an increase in the other (14).

Some works suggest that masturbation does not offer a clear advantage for sexual relationships (15–17). Nonetheless, it has been found that women who masturbate are more likely to have an orgasm during sexual relationships (18), and those who masturbate more frequently describe better sexual experiences in couples and less sexual inhibition (2, 3). Techniques like Directed Masturbation can boost pleasurable stimulation from knowing pleasure points, which improves women's orgasm facility while couples practice sex (19). Therefore, learning to have orgasms by masturbation allows women to adjust and generalize this orgasm response in sexual relationships in couples (20). These results sustain the usefulness of masturbation as the first line of treatment for the Female Orgasmic Disorder (20, 21).

Despite some findings that favor practicing masturbation to improve orgasm capacity, very little evidence exists for the role that the different parameters related to this behavior play in orgasms in the sexual relationships context. Of these parameters, attitude toward masturbation, solitary sexual desire and intensity of the subjective orgasm experience obtained by masturbation stand out. Taking a negative attitude toward masturbation has been associated with feeling guilty and ashamed (22, 23), and also with negative sexual experiences (24). Moreover, lower masturbation frequency, more difficulty to have an orgasm and lower orgasm satisfaction have been observed in those with a more negative attitude toward masturbation (25). Solitary sexual desire (i.e., interest in solitary sexual activity) has been associated with high sexual satisfaction and self-esteem levels in women (2, 4), and has been related to both sexual satisfaction and unsatisfactory sexual functioning in men (26–28). In light of all this, a positive relation between solitary sexual desire and the intensity of the subjective orgasm experience in the solitary masturbation context has been found in a sample made up of men and women (29). Subjective orgasm experience in masturbation has been shown to be capable of distinguishing people with and without difficulties in having an orgasm during sexual relationships (29). Sierra et al. (30) recently observed that masturbation frequency, negative attitude toward masturbation and the subjective orgasmic experience in masturbation are associated with orgasm satisfaction in sexual relationships in people aged over 50 years.

Bearing in mind the relevance of masturbation for sexual health, and its usefulness in the therapeutic context to improve sexual functioning, this study aims to: analyze differences between men and women in different masturbation parameters (i.e., first masturbation experience, current solitary masturbation frequency, negative attitude toward masturbation, solitary sexual desire and subjective orgasm experience); examine their relation, along with age, to orgasm satisfaction in the sexual relationships context. To do so, the following hypotheses are proposed: (1) differences are expected in masturbation parameters between men and women; (2) orgasm satisfaction in sexual relationships is expected to be linked with masturbation parameters (30).

## Methods

### Participants

The sample comprised 1,335 Spanish adults (738 men, 597 women) aged 18–83 years (*M* = 36.91; *SD* = 11.86). The inclusion criteria were: (a) having Spanish nationality; (b) being heterosexual; (c) currently engaging in sexual relationships; (d) having solitary masturbation experience. Table 1 shows the samples' socio-demographic characteristics.

**Table 1:** Sociodemographic characteristics of the participants.

**Variables**	**Total** ***N*** = **1,335**	**Men** ***n*** = **738**	**Women** ***n*** = **597**
Age *M (SD*)	36.91 (11.86)	37.62 (12.43)	36.04 (11.07)
Level of education ***n*** (%)			
Primary education	53 (4.0)	26 (3.5)	27 (4.5)
Secondary education	390 (29.2)	231 (31.3)	159 (26.6)
University degree (ongoing or completed)	892 (66.8)	481 (65.2)	411 (68.9)
Currently have a partner ***n*** (%)			
Yes	988 (74.0)	524 (71.0)	464 (77.7)
No	347 (26.0)	214 (29.0)	133 (22.3)
Praying frequency ***n*** (%)			
Never	989 (74.1)	523 (70.90)	466 (78.1)
Less than once a month	123 (9.2)	67 (9.1)	56 (9.4)
Once a month	7 (0.5)	6 (0.8)	1 (0.2)
A few times a month	54 (4.0)	32 (4.3)	22 (3.7)
Once a week	8 (0.6)	3 (0.4)	5 (0.8)
A few times a week	57 (4.3)	34 (4.6)	23 (3.9)
Once a day	60 (4.5)	41 (5.6)	19 (3.2)
More than once a day	37 (2.8)	32 (4.3)	5 (0.8)

### Measures

Background questionnaire. This instrument collects data about sex, age, level of education, nationality, sexual orientation, partner relationship, frequency of prayer, age when the first masturbation experience occurred and masturbation frequency.

The Spanish version of the Negative Attitudes Toward Masturbation Inventory (NATMI) (25, 31). It evaluates negative attitudes toward masturbation with 10 items (e.g., “I feel guilty about masturbating”) answered on a 5-point Likert-type scale: 1 (Not at all true for me) to 5 (Extremely true for me). Higher scores indicate a more negative attitude toward masturbation. It has a high internal consistency (alpha ordinal) of 0.95, and presents suitable evidence for construct and discriminant validity with other psychosexual variables and sexual functioning. In this sample, the ordinal alpha coefficient was 0.91.

The Solitary Sexual Desire subscale from the Spanish version of the Sexual Desire Inventory (SDI) (28, 32). It consists of four items (e.g., “How strong is your desire to engage in sexual behavior by yourself?”) and measures interest in solitary sexual activity using different Likert response scales depending on the item (e.g., from 0 = No desire to 8 = Strong desire). Higher scores show more solitary desire. It presents good internal consistency (Cronbach's α higher than 0.90) and evidence for external validity. Cronbach's alpha in the present study was 0.91.

The Spanish version of the Orgasm Rating Scale (ORS) (33) adapted to the solitary masturbation context by Cervilla et al. (29). It assesses the subjective orgasm experience in the solitary masturbation context (during any sexual activity performed alone) with 25 adjectives distributed on four dimensions: Affective, Sensory, Intimacy, and Rewards. Items are answered on a 6-point Likert-type scale: 0 (Does not describe it at all) to 5 (Describes it perfectly). Higher scores indicate more intensity in the subjective orgasm experience. Its internal consistency reliability is good and ranges from 0.71 to 0.95. It adequately evidences validity, provided by its measures. In our study, the ordinal alphas for the different subscales were: 0.93 for Affective, 0.94 for Sensory, 0.72 for Intimacy and 0.89 for Rewards.

The Spanish version of the Arizona Sexual Experience Scale (ASEX) (34) of Sánchez-Fuentes et al. (35). It consists of five items that assess general sexual functioning (sexual desire, arousal, erection for men/lubrication for women, orgasm, and orgasm satisfaction) in the last 7 days in the sexual relationship context. It uses a Likert-type scale from 1 (hypofunction) to 6 (hyperfunction). It presents good internal consistency (Cronbach's alpha of 0.81 in men, 0.79 in women) and evidences validity. The orgasm-related item referring to orgasm satisfaction was taken into account. Its score was inverted, so higher scores evidenced more orgasm satisfaction.

### Procedure

Data collection was conducted by distributing a survey using LimeSurvey, which was promoted by paying to Facebook (900€) from 23 December 2019 to 15 March 2020 by adults from Spain. In order to improve the representativeness of the sample, the promotion targeted both men and women from different age groups. Online assessments are normally used to evaluate sexual behaviors (1, 36, 37). Previous studies have confirmed that there are no differences between online and paper-and-pen methods (38, 39). To avoid automatic or fraudulent responses, IP was controlled and a CAPTCHA was used. In addition, responses were carefully examined to rule out non conclusive or abnormal cases. Participation was voluntary, and both anonymity and confidentiality of responses were guaranteed. There was no compensation for taking part in the study. All the participants received informed consent with the study aim before responding. This research was approved by the Ethics Committee of Human Research of the University of Granada.

### Statistical analysis

A cross-sectional correlational study is proposed. First, missing values were imputed using a random forest algorithm by considering the associated variables. To examine differences in the masturbation parameters between men and women, a MANCOVA was applied for first masturbation experience (age), current solitary masturbation frequency (“Never,” “Less than once a month,” “Once a month,” “A few times a month,” “Once a week,” “A few times a week,” “Once a day” and “More than once a day”), negative attitude toward masturbation, solitary sexual desire and subjective orgasm experience caused by masturbation, and by taking into account these covariates: age, level of education (“Primary Education,” Secondary Education” and “University degree–ongoing or completed-”), having a partner (yes or no) and frequency of prayer (similar to the masturbation frequency). Considering the differences found by sex, the subsequent analyses were presented separately for men and women. The capacity of the masturbation parameters to explain orgasm satisfaction was examined by multiple linear regression using the enter method.

The R^®^ environment was employed (version 3.6.3) (40) with its RStudio^®^ interface (version 1.2.5042) (41). For missing value imputations, the missForest package was used (version 1.4) (42). For the ordinal alpha, the Psych package was applied (version 1.9.12.31) (43). The other statistical analyses were performed with SPSS v.22.

## Results

### Sex differences in the masturbation parameters

The significant multivariate covariates were age [Wilk's lambda = 0.87; *F*_(8, 1, 322)_ = 24.49, *p* < 0.001; η^2^ = 0.129], having a partner [Wilk's lambda = 0.94; *F*_(8, 1, 322)_ = 10.91, *p* < 0.001; η^2^ = 0.062] and frequency of prayer [Wilk's lambda = 0.96; *F*_(8, 1, 322)_ = 7.42, *p* < 0.001; η^2^ = 0.04]. Sex had a main effect on the masturbation parameters [Wilk's lambda = 0.77; *F*_(8, 1, 322)_ = 48.91, *p* < 0.001; η^2^ = 0.23]. The intersubject effect on these indicators is shown in Table 2.

**Table 2:** Effects of sex on masturbation-related indicators.

**Variables** ***M (DT*****)**	**Males** ***n*** = **738**	**Females** ***n*** = **597**	*F*_(1, 1, 329)_	***p***	***Cohen's d***
First masturbation experience	12.60 (2.03)	15.13 (5.92)	140.51	<0.001	0.60
Current frequency of solitary masturbation	3.17 (0.94)	2.50 (0.96)	185.11	<0.001	0.70
Negative attitude toward masturbation	13.03 (3.09)	13.13 (2.32)	1.91	0.167	-
Solitary sexual desire	21.59 (5.59)	19.75 (6.28)	31.96	<0.001	0.31
Subjective orgasm experience-Affective	25.66 (4.84)	27.05 (3.85)	31.69	<0.001	0.31
Subjective orgasm experience-Sensory	33.65 (15.74)	39.17 (15.15)	47.75	<0.001	0.36
Subjective orgasm experience-Intimacy	7.62 (3.64)	8.19 (3.71)	12.48	<0.001	0.15
Subjective orgasm experience-Rewards	11.34 (3.36)	11.56 (3.59)	1.82	0.177	-

### Regression models

For men, a significant model was obtained that explained orgasm satisfaction in sexual relationships [*F*_(9, 728)_ = 13.01; *p* < 0.001]. Current solitary masturbation frequency (β = −0.10) and the Affective dimension of orgasm (β = 0.36) explained 13% of variance (See Table 3). The model was also significant for women [*F*_(9, 587)_ = 8.88; *p* < 0.001] and explained 11% of orgasm satisfaction from age (β = 0.09), negative attitude toward masturbation (β = −0.12), solitary sexual desire (β = 0.19) and the Affective dimension of orgasm (β = 0.24) (See Table 4).

**Table 3:** Multiple regression models for orgasmic satisfaction in men.

**Predictors**	***B***	***SE***	β	**95%** ***CI***	***t***	***p***	***R** ^2^*	**VIF**
Orgasmic satisfaction							0.13	
Age	0.00	0.00	0.01	−0.00, 0.01	0.03	0.974		1.23
First masturbation experience	−0.01	0.02	−0.03	−0.05, 0.02	−0.88	0.379		1.08
Current frequency of solitary masturbation	−0.10	0.04	−0.10	−0.19, −0.01	−2.22	0.027		1.79
Negative attitude toward masturbation	−0.01	0.01	−0.05	−0.04, 0.01	−1.31	0.191		1.15
Solitary sexual desire	0.01	0.01	0.08	−0.00, 0.03	1.77	0.076		1.92
Subjective orgasm experience-Affective	0.07	0.01	0.36	0.05, 0.08	7.70	<0.001		1.85
Subjective orgasm experience-Sensory	−0.00	0.00	−0.05	−0.01, 0.00	−1.09	0.275		1.93
Subjective orgasm experience-Intimacy	0.00	0.01	0.01	−0.02, 0.03	0.32	0.746		1.70
Subjective orgasm experience-Rewards	−0.01	0.01	−0.03	−0.03, 0.01	−0.78	0.438		1.40

*B, non-estandardized beta; SE, standard error; β, standardized beta; 95% CI, 95% confidence interval; VIF, variance inflation factor*.

**Table 4:** Multiple regression models for orgasmic satisfaction in women.

**Predictors**	***B***	***SE***	β	**95% CI**	***t***	***p***	***R** ^2^*	**VIF**
Orgasmic satisfaction							0.11	
Age	0.01	0.00	0.09	0.00, 0.02	2.08	0.038		1.21
First masturbation experience	0.01	0.01	0.04	−0.01, 0.02	1.01	0.314		1.10
Current frequency of solitary masturbation	−0.07	0.06	−0.07	−0.18, 0.04	−1.27	0.203		1.88
Negative attitude toward masturbation	−0.05	0.02	−0.12	−0.09, −0.01	−2.80	0.005		1.15
Solitary sexual desire	0.03	0.01	0.19	0.01, 0.05	3.43	0.001		2.11
Subjective orgasm experience-Affective	0.06	0.01	0.24	0.04, 0.09	4.91	<0.001		1.65
Subjective orgasm experience-Sensory	−0.00	0.00	−0.03	−0.01, 0.00	−0.52	0.606		1.77
Subjective orgasm experience-Intimacy	0.00	0.01	0.01	−0.03, 0.03	0.12	0.906		1.68
Subjective orgasm experience-Rewards	−0.02	0.01	−0.08	−0.05, 0.00	−1.70	0.090		1.46

*B, non-estandardized beta; SE, standard error; β, standardized beta; 95% CI, 95% confidence interval; VIF, variance inflation factor*.

## Discussion

Masturbation is a sexual behavior that is contemplated to deal with sexual dysfunctions, especially orgasm difficulties (44–46). Justifying the use of masturbation in sexual therapy lies in the relation between this behavior and orgasm in sexual relationships. This is why the present study analyzes the relation between different masturbation parameters in men and women (i.e., first masturbation experience, current solitary masturbation frequency, negative attitude toward masturbation, solitary sexual desire and subjective orgasm experience) with orgasm satisfaction in sexual relationships. The results show differences between men and women in the masturbation parameters, and also in the role that these parameters play in explaining orgasm satisfaction in the sexual relationships context.

The first hypothesis is backed by significant differences between men and women in the different masturbation parameters. We observe that men's first masturbation experience took place at an earlier age than it did in women, whose finding coincides with previous studies (1, 2, 25, 30, 47). Traditional sexual socialization could favor more permissiveness in men and more guilty feelings associated with women practicing masturbation (48). In turn, the differences found in solitary masturbation frequency coincide with previous works in the literature, and a more frequent masturbation frequency observed for men (25, 49, 50). Attitude to the sexual double standard (i.e., the distinct evaluation made of sexual behavior depending on whether it is practiced by a man or a woman) could explain these differences given the greater sexual freedom or permissiveness that men have been traditionally conferred than women (38). Alternative considerations have also been applied to explain these differences in association with hormone levels (51).

It is worth mentioning that no differences have been found in negative attitude toward masturbation between men and women. The fact that such differences are lacking could be related to an increasingly more positive change of attitude in both men and women, as observed in other attitudes like erotophilia (52). These results contradict those recently obtained in older people and reported by Sierra et al. (30), who indicate that men older than 50 years take a more negative attitude toward masturbation than women of a similar age. This could indicate younger generations' positive attitude toward masturbation. This question reflects the need to further study in-depth attitudes toward masturbation and the factors related to it to better understand this matter (25). Regarding differences in solitary sexual desire, the highest level found for men is consistent with previous works that report similar results (27, 28, 53, 54). This is congruent with those studies showing a close association between masturbation and solitary sexual desire (55).

On subjective orgasm experience in solitary masturbation, and in line with the results obtained by previous studies that have examined the subjective orgasm experience in the heterosexual relationships context (36, 56), women report greater intensity than men, except on the Reward dimension, which has also been shown for the gay population (57). To explain differences in orgasm intensity between men and women, women have been proposed to better localize orgasms anatomically (56), which would be associated with perceiving greater intensity (58). It has also been indicated that women could have a bigger repertoire to describe their orgasm sensations (57, 59). Regarding the differences in their dimensions, not finding discrepancies would be expected on the Rewards dimension, which is made up of the items “peaceful,” “relaxing,” and “soothing,” because both men and women have pointed out that relaxing is one of the main reasons to masturbate (13, 60, 61).

Regarding our second hypothesis, orgasm satisfaction in the sexual relationships context is explained in both men and women by some masturbation parameters. In the model for men and women, the Affective dimension of the subjective orgasm experience during masturbation significantly and positively explains orgasm satisfaction in the sexual relationships context. Former findings stress the importance of the Affective dimension of the subjective orgasm experience for the sexual relationships context, especially for women (62). So it might seem logical to think that this could be the case in the masturbation context where this dimension is more important for explaining orgasm satisfaction in sexual relationships.

Apart from the orgasm Affective dimension in the men's model, higher solitary masturbation frequency is also associated with lower orgasm satisfaction. These results might appear to contradict works that have described how frequency is associated with more consistent orgasms (7). However, in line with previous studies (30, 52), this association might be explained by the compensatory model of masturbation; that is, masturbation serving as a substitute of unsatisfactory sexual relationships. Therefore, lower orgasm satisfaction in the sexual relationships context might be expected to be compensated by higher masturbation frequency (26, 63).

In women, apart from the Affective dimension of orgasm, age and solitary sexual desire are positively associated, and attitude toward masturbation is negatively associated, with orgasm satisfaction in sexual relationships. The positive association of age would be expected because former works inform about a higher orgasm pleasure level with increasing age (18, 61). Moreover, the positive relation between sexual desire and sexual functioning has been well-described (27, 28, 64). In fact solitary sexual desire is associated with higher masturbation frequency (1, 55), which might imply more self-erotic experiences and sexual self-knowledge (3). Finally, the fact that negative attitude toward masturbation is related to lower orgasm satisfaction is consistent with previous works (25, 65). This attitude has been associated with lower masturbation frequency (25), which might imply fewer opportunities for both sexual response self-knowledge and the associated pleasure points (7, 19).

Some differences between the models for men and women are worth stressing. The positive effect of age is only observed in women. This suggests that women benefit from enjoying more orgasm satisfaction as they age to a certain extent. Despite a negative association between age and orgasm capacity having been previously described (36, 66), these results are consistent with some findings which reveal that women need time to interiorize a more positive relation with masturbation due to the stigmatization that their engagement in such behavior might imply (2). This suggests that the positive effects of masturbation could increase as women age. Besides, solitary masturbation frequency only has a significant effect on men, which falls in line with former results which point out that masturbation frequency in women is not significantly associated with orgasm outcomes (18). As higher masturbation frequency in men is associated with lower orgasm satisfaction in sexual relationships, it would be coherent to think that solitary sexual desire plays no relevant role to explain men's orgasm satisfaction. Finally, the differences observed in the models of men and women fall in line with the previous literature, which emphasizes how women's orgasm is associated with more variables than it is for men (56, 57, 67).

This study has its limitations, which must be taken into account to generalize its results. The study sample was formed by incidental non probabilistic sampling over social networks and only included the heterosexual population. The cross-sectional correlational experimental design and the performed statistical analyses do not allow for causality relations. So, it may be need longitudinal studies to have a deep approach about the relationship between masturbation and sexual relationships. Different parameters of masturbation could be taken into account in future studies, such as the duration of masturbation, the use of erotic toys, the techniques used or the consumption of pornography. Notwithstanding, the findings are believed relevant for its contribution to the study of masturbation and orgasm satisfaction in the sexual relationships context.

## Conclusion

The obtained results confirm the differences between men and women in the masturbation parameters and their role to explain orgasm satisfaction in sexual relationships. The Affective dimension of the subjective orgasm experience during solitary masturbation is stressed as a common variable for both men and women to explain orgasm satisfaction in sexual relationships. More masturbation parameters associated with orgasm satisfaction are observed in women than men. These findings suggest that the relation between solitary masturbation and sexual relationships is a complex one. Masturbation in men could be a substitute for the satisfaction not achieved with orgasm in sexual relationship; in women, the negative attitude toward this behavior would be associated with lower orgasmic satisfaction, and a greater solitary sexual desire could promote more sexual self-knowledge. So it is important to consider these results to look more closely at the association between both sexual behaviors, and to further consolidate the usefulness of solitary masturbation in sexual therapy. Therefore, solitary masturbation is an available resource that should also be promoted in the community context as it can improve the sexual health of the population.

## Data availability statement

The raw data supporting the conclusions of this article will be made available by the authors, without undue reservation.

## Ethics statement

The studies involving human participants were reviewed and approved by the Ethics Committee of Human Research of the University of Granada. The patients/participants provided their written informed consent to participate in this study.

## Author contributions

The concept and design: JCS. Acquisition, analysis, interpretation of data, drafting of the manuscript, critical revision of the manuscript, and statistical analysis: JCS and OC. All authors contributed to the article and approved the submitted version.

## Funding

This study has been funded by the Ministerio de Ciencia, Innovación y Universidades through the Research Project RTI2018-093317-B-I00 and the Bursary FPU18/03102 for University Professor Training as part of OC's thesis (Psychological Doctoral Programme B13 56 1; RD 99/2011).

## Conflict of interest

The authors declare that the research was conducted in the absence of any commercial or financial relationships that could be construed as a potential conflict of interest.

## Publisher's note

All claims expressed in this article are solely those of the authors and do not necessarily represent those of their affiliated organizations, or those of the publisher, the editors and the reviewers. Any product that may be evaluated in this article, or claim that may be made by its manufacturer, is not guaranteed or endorsed by the publisher.
